# Supplementary material for: Hyperuniformity with no fine tuning in sheared sedimenting suspensions
Source: Nat Commun. 2018 Jul 19;9:2836. doi: 10.1038/s41467-018-05195-4 (PMC6053396; doi:10.1038/s41467-018-05195-4)
Supplement: Supplementary file 1 — Supplementary Information [file 41467_2018_5195_MOESM1_ESM.pdf]

Supplementary Information for
“Hyperuniformity with no fine tuning in sheared sedimenting suspensions”

Wang et al.

I. SUPPLEMENTARY FIGURES

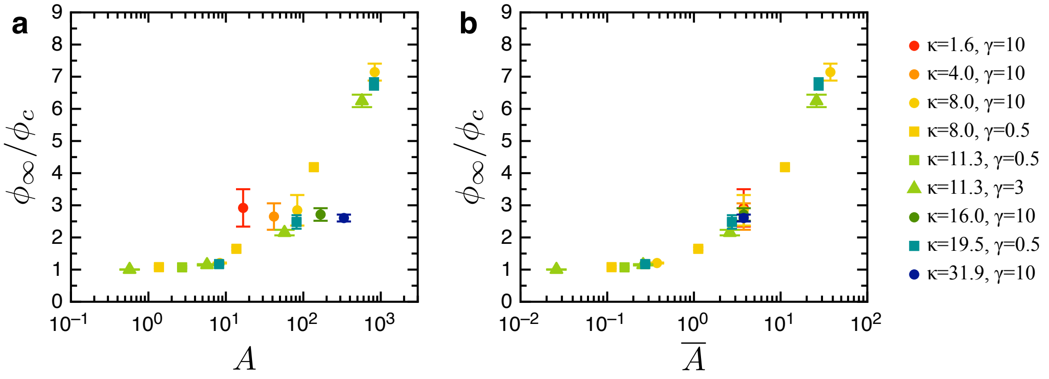

SUPPLEMENTARY FIG. 1. **Results for multiple kicks.** Scaled steady-state concentration in a suspension sedimenting at velocity $10^{-4} < v_s < 10^{-1}$, sheared at strain amplitude γ. We use $N = 1000$, $\epsilon = 0.5$, and $1.6 < \kappa < 31.9$. The diffusion constant D is set to the value obtained by Corté *et al.* [1] (i.e., $D = 0.0414$). **(a)** The data only approximately collapse with $A = (\pi/\phi_c)^{3/2} d^3 \kappa^2 v_s / 32D$, the scaling proposed by Ref. [1]. **(b)** The data collapse cleanly when plotted versus $\overline{A} = \pi d^2 \kappa v_s / 16 \phi_c D$.

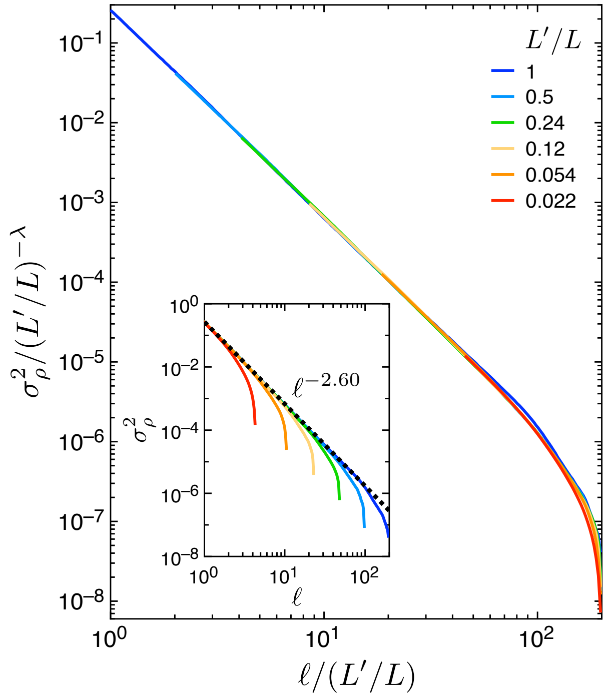

SUPPLEMENTARY FIG. 2. **Effect of system size on variance of the number density.** *Inset:* Variance of the number density, σ_ρ^2, versus ℓ for a square system of size $L = 200$ and subsystems of size $L' < L$. The largest system has $N = 10186$, $\phi = 0.2$, and is sheared cyclically at amplitude $\gamma = 3.01 \approx \gamma_c$ until it reaches a reversible state. We thus obtain a hyperuniform scaling with $\lambda = 2.60$. *Main:* By scaling the x and y axes, the data are collapsed onto a master curve, which falls off as ℓ approaches the system size.

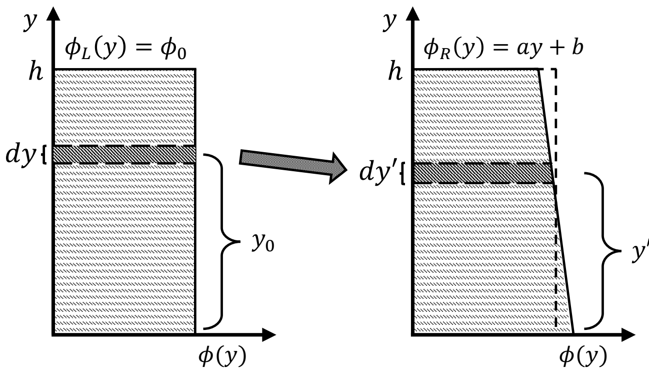

SUPPLEMENTARY FIG. 3. **Distorting a uniform system to impose a constant vertical concentration gradient.** *Left:* Concentration profile, $\phi_L(y)$, versus vertical position, y (where the dependent variable is on the x axis so that vertical axis is oriented as in the simulation). The system has a uniform distribution along the y axis with a constant concentration ϕ_0. *Right:* Target concentration profile of the form $\phi_R(y) = ay + b$. The shaded region at position y with thickness dy is mapped to position y' with thickness dy' that conserves its mass. Note that the height, h, of the initial and final profiles is constrained to be the same.

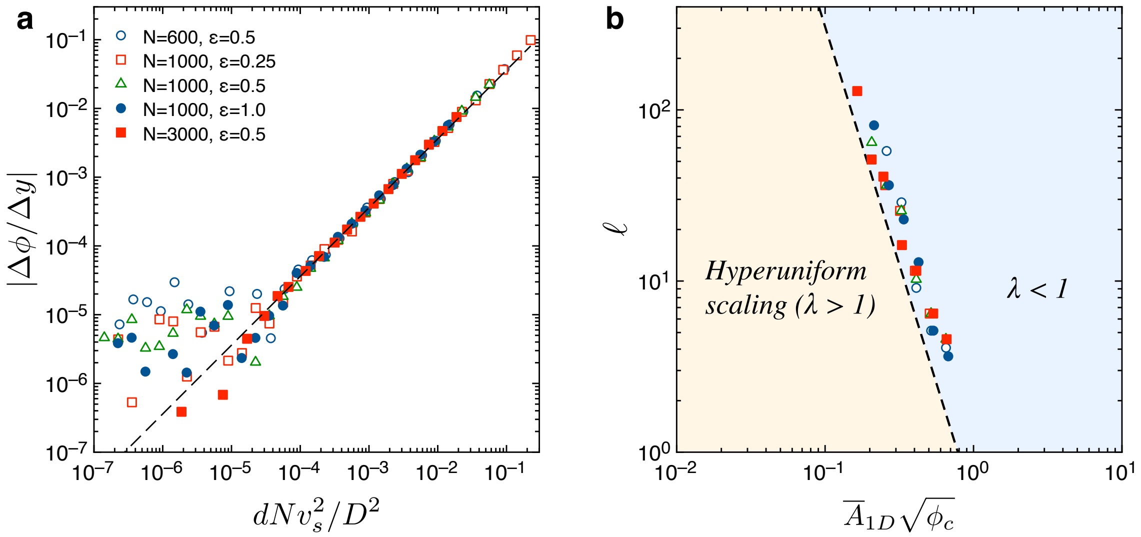

SUPPLEMENTARY FIG. 4. **One-dimensional model results. (a)** Magnitude of the vertical concentration gradient, $|\Delta\phi/\Delta y|$ versus dNv_s^2/D^2, in the steady state. Data are from fits to the middle 60% of the concentration profile, averaged over 20 systems. Dashed line: Predicted scaling with fitted numerical prefactor given by Supplementary Eq. 5. **(b)** Phase diagram. Symbols: Lengthscale $\ell_{\rm H}$ where the local scaling exponent λ of the variance $\sigma_\rho^2(\ell)$ is shallower than 1. As in our 2D systems, we measure the variance over the bottom 99% of particles, and we consider windows of size $\ell < h_\infty/5$ to avoid system size effects. Dashed line: Phase boundary from our theory with no free parameters, Supplementary Eq. 6. As in 2D, hyperuniform scaling emerges below a finite threshold value of $\overline{A}\sqrt{\phi_c}$ and extends to longer lengthscales at smaller $\overline{A}\sqrt{\phi_c}$.

II. SUPPLEMENTARY NOTES

Supplementary Note 1: Dependence of self-organized criticality on collision rule. The simulation model for cyclically-sheared viscous suspensions that we use in this work was originally developed by Ref. [2] to study self-organized reversible states. Variants of this model have been studied in recent years. Reference [3] studied a wide range of driving and collision rules to test for the robustness of results on memory formation; Refs. [4, 5] used isotropic swelling in place of shear as a simpler method for studying the critical transition, and Refs. [3, 6] used center-of-mass conserving collisions to suppress long-range diffusion.

Here we probe one aspect of these kinematics in order to make a more precise comparison to previous results [1]. In a dense portion of the sample, a particle can encounter multiple other particles during a single cycle. In the present work we give just one kick to such a particle, whereas Corté *et al.* [1] gave one kick for each particle encountered, which increases diffusion rates in dense regions.

We find that the scaling for obtaining the critical concentration under sedimentation does not change between these two models. Supplementary Fig. 1 shows ϕ_∞/ϕ_c for simulations we performed with the "multiple kick" rule. The data only approximately collapse when plotted versus A, but they collapse cleanly when plotted versus $\overline{A}$. To contrast the two expressions, in 5 of these simulations we kept the product κv_s constant while varying κ from 1.6 to 31.9 (with $\gamma = 10$), so that $\overline{A} \propto \kappa v_s$ is fixed but $A \propto \kappa^2 v_s$ varies. Those points show that the data are better collapsed by $\overline{A}$, as we found for single kicks in the main text. This test also serves as a further systematic check on our results, as the two simulation codes were written independently by two of us (J.W. and J.D.P.).

Supplementary Note 2: Finite size effects. In Fig. 4 in the main text, the variance of the number density is observed to fall off rapidly at large ℓ. This occurs when the window size, ℓ, is a significant fraction of the narrowest system dimension, causing the sampling windows to overlap. The samples are therefore not statistically independent, so the variation in the number density is suppressed. This effect has been investigated previously for small variations in system size [7].

Here we study this effect by probing a large range of system sizes in simulations without sedimentation. We produce hyperuniform distributions of particles by shearing a square system of side length L near the critical amplitude γ_c. As in the main text, we measure the variance of the number density, which decays as $\ell^{-\lambda}$ with $\lambda \approx 2.60$. We then cut subsystems of side length $L' < L$ out of the original system, and we measure the variance in the same manner. This is repeated for a total of 6 system sizes, with ratios L'/L from 0.022 to 1. Each curve is averaged over 42 systems to suppress noise.

The results are shown in the inset to Supplementary Fig. 2. Each curve follows the same scaling with ℓ and falls off rapidly when ℓ approaches L'. We shift the curves by rescaling the x axis by L'/L and the y axis by $(L'/L)^{-2.60}$. Remarkably, all the data fall onto a single master curve, which shows that the effect is only sensitive to the ratio ℓ/L.

Supplementary Note 3: Introducing a concentration gradient into a hyperuniform system. Here we derive the mapping used in Fig. 4c of the main text to produce artificial systems with constant concentration gradients. As stated in the main text, the mapping is uniquely determined by requiring a concentration map $\phi_0 \to \phi(y) = \phi_0 + |\partial\phi/\partial y|(h - 2y)/2$ on a continuum system with uniform concentration ϕ_0, where $0 < y < h$.

We denote the initial uniform distribution by $\phi_L(y) = \phi_0$ and the target distribution by $\phi_R(y) = ay + b$, as drawn in Supplementary Fig. 3. The total area under these curves must be identical, so $b = \phi_0 - ah/2$, where h is the height of the system. The mapping must also conserve mass; for a

region from the bottom of the system up to a height y, we have:

$$\int_0^y \phi_L(y)dy = \int_0^{y'} \phi_R(y)dy, \tag{1}$$

where the point at height y maps to height y'. Plugging in the expressions for ϕ_L and ϕ_R and performing the integral, we get:

$$\phi_0 y = \frac{1}{2}ay'^2 + \left(\phi_0 - \frac{ah}{2}\right)y'. \tag{2}$$

Solving for y', we retrieve the mapping:

$$y' = \frac{h}{2} - \frac{\phi_0}{a} - \sqrt{\frac{2\phi_0 y}{a} + \left(\frac{h}{2} - \frac{\phi_0}{a}\right)^2}, \tag{3}$$

where $a = \partial\phi/\partial y$ is the target vertical concentration gradient.

Supplementary Note 4: One-dimensional model. We can predict the loss of hyperuniform scaling in the 1D system by tailoring the arguments in the main text to this dimension. First, the nondimensional $\overline{A}_{\mathrm{1D}}$ is given by:

$$\overline{A}_{\mathrm{1D}} = \frac{1}{2}\frac{dNv_s}{\phi_c D}. \tag{4}$$

Equation 5 in the main text (for the effect of a concentration gradient on the variance of the number density) is modified to: $\sigma_\rho^2(\ell)_{\mathrm{grad}} = (\partial\phi/\partial y)^2(h-\ell)^2/12$. Equation 7 in the main text (for the scaling for vertical concentration gradients) is modified to:

$$\left|\frac{\Delta\phi}{\Delta y}\right| \approx 0.36\frac{dNv_s^2}{D^2}, \tag{5}$$

where we obtain the numerical prefactor by fitting to the data shown in Supplementary Fig. 4a.

Adding the variance due to the vertical concentration gradient to the variance in the critical state without sedimentation, we get for the total variance in 1D: $\sigma_\rho^2(\ell)_{\mathrm{total}} \approx \sigma_\rho^2(\ell)_c + 0.17\overline{A}_{\mathrm{1D}}^4\phi_c^2$. We then solve for the lengthscale ℓ_{H} where the local scaling exponent is equal to $\lambda = 1$, where we use $\sigma_\rho^2(\ell)_c \approx 0.15\ell^{-1.44}$ from our measurements in 1D (shown in Fig. 4b in the main text). This computation yields:

$$\ell_{\mathrm{H}} \approx 0.51(\overline{A}_{\mathrm{1D}}\sqrt{\phi_c})^{-2.78}. \tag{6}$$

We plot the 1D phase diagram in Supplementary Fig. 4b, where the data are obtained in the same fashion as in 2D. The prediction is in good agreement with the data.

III. SUPPLEMENTARY REFERENCES

[1] L. Corté, S. J. Gerbode, W. Man, and D. J. Pine, Phys. Rev. Lett. **103**, 248301 (2009).
[2] L. Corté, P. M. Chaikin, J. P. Gollub, and D. J. Pine, Nat. Phys. **4**, 420 (2008).
[3] N. C. Keim, J. D. Paulsen, and S. R. Nagel, Phys. Rev. E **88**, 032306 (2013).
[4] E. Tjhung and L. Berthier, Phys. Rev. Lett. **114**, 148301 (2015).
[5] E. Tjhung and L. Berthier, J. Stat. Mech. Theory Exp. **2016**, 033501 (2016).
[6] D. Hexner, P. Chaikin, and D. Levine, Proc. Natl. Acad. Sci. U.S.A. **114**, 4294 (2017).
[7] R. Dreyfus, Y. Xu, T. Still, L. A. Hough, A. Yodh, and S. Torquato, Phys. Rev. E **91**, 012302 (2015).
