# Supplementary material for: Hyperuniformity with no fine tuning in sheared sedimenting suspensions
Source: Nat Commun. 2018 Jul 19;9:2836. doi: 10.1038/s41467-018-05195-4 (PMC6053396; doi:10.1038/s41467-018-05195-4)
Supplement: Supplementary file 2 — Description of Additional Supplementary Files [file 41467_2018_5195_MOESM2_ESM.pdf]

Description of Additional Supplementary Files

File Name: Supplementary Movie 1

Description: Two-dimensional simulation with $\overline{A}$=1.2 (where N=2420, $v_s=10^{-3}$, γ=3, κ=24.2, and ε=0.5), strobed every 500 cycles for 9×10^4 cycles. Strong vertical concentration gradients occur.

File Name: Supplementary Movie 2

Description: Two-dimensional simulation with $\overline{A}$=0.12 (where N=2420, $v_s=10\text{-}^{4}$, γ=3, κ=24.2, and ε=0.5), strobed every 500 cycles for 2×10^5 cycles. The particle concentration is approximately constant as a function of height.

File Name: Supplementary Movie 3

Description: One-dimensional simulation with $\overline{A}_{1D}$=0.15 (where N=100, $v_s=10\text{-}^{4}$, and ε=0.5), strobed every 200 cycles for 4×10^5 cycles.
